# Development of a locator device usability scale for persons with dementia at risk of getting lost

**DOI:** 10.1093/geroni/igab046.2601

**Published:** 2021-12-17

**Authors:** Noelannah Neubauer, Christa Spenrath, Serrina Philip, Christine Daum, Lili Liu, Antonio Miguel-Cruz

**Affiliations:** 1 University of Waterloo, Waterloo, Ontario, Canada; 2 University of Alberta, Edmonton, Alberta, Canada; 3 University of Waterloo, Edmonton, Alberta, Canada

## Abstract

There is an increasing number of persons living with dementia who live alone. Recent COVID-19 pandemic restrictions have resulted in more persons receiving care remote through information and communication technologies. Locating technologies can be a tool to help care partners monitor their loved ones living with dementia. These devices can also mitigate risks associated with going missing, by reducing time for search and returning the lost person home safely. However, there is no clear, standardized approach to assess the usability of these devices. The purpose of this study was to develop a locator device usability scale for persons living with dementia at risk of getting lost. A two-phase study that utilized a multi-method design and included participatory and iterative strategies was conducted. In the first phase, an item pool was generated through online focus groups with service providers, technology developers, care partners and persons living with dementia. The second phase refined the item pool using an online survey and online focus groups with the same stakeholder groups. Five overarching categories were identified as important for the usability of locating device: features, inclusivity, simplicity, aesthetic appeal, and ethics. Participants identified the need for multiple versions of the usability scale including one specifically for persons living with dementia. The newly developed locator device usability scale can enhance the acceptance of these devices, thereby supporting remote caregiving and promote the safety and autonomy of persons living with dementia.

